# Lifestyle Medicine Reimbursement: A Proposal for Policy Priorities Informed by a Cross-Sectional Survey of Lifestyle Medicine Practitioners

**DOI:** 10.3390/ijerph182111632

**Published:** 2021-11-05

**Authors:** Kelly J. Freeman, Meagan L. Grega, Susan M. Friedman, Padmaja M. Patel, Ron W. Stout, Thomas M. Campbell, Michelle L. Tollefson, Liana S. Lianov, Kaitlyn R. Pauly, Kathryn J. Pollard, Micaela C. Karlsen

**Affiliations:** 1 Department of Member Engagement & Administration, American College of Lifestyle Medicine, Chesterfield, MO 63006, USA; kpauly@lifestylemedicine.org; 2 Health Policy & Management, Indiana University Richard M. Fairbanks School of Public Health, Indianapolis, IN 46202, USA; 3 Department of Lifestyle Medicine, Kellyn Foundation, Tatamy, PA 18015, USA; meagan@kellynfoundation.org; 4 School of Medicine and Dentistry, Department of Medicine, University of Rochester, Rochester, NY 14642, USA; Susan_Friedman@URMC.Rochester.edu; 5 American College of Lifestyle Medicine, Chesterfield, MO 63006, USA; padmaja.patel@midland-memorial.com (P.M.P.); friends@myhappyavatar.com (L.S.L.); 6 Lifestyle Medicine Center, Midland Health, Midland, TX 79703, USA; 7 Ardmore Institute of Health, Ardmore, OK 73401, USA; ron.stout@ardmoreinstituteofhealth.org; 8 Department of Family Medicine, University of Rochester Medical Center, Rochester, NY 14642, USA; thomas_campbell@urmc.rochester.edu; 9 Department of Health Professions, Lifestyle Medicine Program, Metropolitan State University of Denver, Denver, CO 80204, USA; mtollef2@msudenver.edu; 10 Global Positive Health Institute, Sacramento, CA 95825, USA; 11 Department of Research, American College of Lifestyle Medicine, Chesterfield, MO 63006, USA; kpollard@lifestylemedicine.org (K.J.P.); mkarlsen@lifestylemedicine.org (M.C.K.)

**Keywords:** lifestyle medicine, reimbursement, quality measures, healthcare policy, intensive therapeutic lifestyle changes, person-centered care

## Abstract

Lifestyle medicine (LM) is a rapidly emerging clinical discipline that focuses on intensive therapeutic lifestyle changes to treat chronic disease, often producing dramatic health benefits. In spite of these well-documented benefits of LM approaches to provide evidence-based care that follows current clinical guidelines, LM practitioners have found reimbursement challenging. The objectives of this paper are to present the results of a cross-sectional survey of LM practitioners regarding lifestyle medicine reimbursement and to propose policy priorities related to the ability of practitioners to implement and achieve reimbursement for these necessary services. Results from a closed, online survey in 2019 were analyzed, with a total of *n* = 857 included in this analysis. Results were descriptively analyzed. This manuscript articulates policy proposals informed by the survey results. The study sample was 58% female, with median age of 51. A minority of the sample (17%) reported that all their practice was LM, while 56% reported that some of their practice was LM. A total of 55% of practitioners reported not being able to receive reimbursement for LM practice. Of those survey respondents who provided an answer to the question of what would make the practice of LM easier (*n* = 471), the following suggestions were offered: reimbursement overall (18%), reimbursement for more time spent with patients (17%), more support from leadership (16%), policy measures to incentivize health (13%), education in LM for practitioners (11%), LM-specific billing codes and billing knowledge along with better electronic medical record (EMR) capabilities and streamlined reporting/paperwork (11%), and reimbursement for the extended care team (10%). Proposed policy changes focus on three areas of focus: (1) support for the care process using a LM approach, (2) reimbursement emphasizing outcomes of health, patient experience, and delivering person-centered care, and (3) incentivizing treatment that produces disease remission/reversal. Rectifying reimbursement barriers to lifestyle medicine practice will require a sustained effort from health systems and policy makers. The urgency of this transition towards lifestyle medicine interventions to effectively address the epidemic of chronic diseases in a way that can significantly improve outcomes is being hindered by current reimbursement policies and models.

## 1. Introduction

The majority of guidelines for treatment and management of chronic disease offer lifestyle changes [1] as a first-line intervention, including guidelines for cardiovascular disease [2], diabetes [3,4], and hypertension [5]. These guidelines make strong and specific recommendations regarding therapeutic lifestyle interventions, including adopting healthy dietary patterns consisting of primarily whole-food, plant-based nutrition and optimizing the amount, consistency, and intensity of exercise. However, many challenges exist with respect to implementing lifestyle interventions in the predominant healthcare model. Lifestyle medicine is a clinical discipline in which practitioners and the entire healthcare team treat many common non-communicable chronic diseases using health behavior change as the foundation of care. The American College of Lifestyle Medicine (ACLM) defines lifestyle medicine (LM) as the use of evidence-based lifestyle therapeutic interventions—including a whole-food, plant-predominant eating pattern, regular physical activity, restorative sleep, stress management, avoidance of risky substances, and positive social connection—as a primary modality, delivered by clinicians trained and certified in this specialty, to prevent, treat, and often reverse chronic disease. Risky substances can include anything harmful or addictive, such as nicotine, alcohol, and opioids. LM is applicable in almost every realm of healthcare, including maternal, pediatric, and family practice, as well as specialties such as cardiology, internal medicine, and endocrinology, within outpatient and inpatient services. LM can also be practiced at a specialty level, where patients are referred to a healthcare provider or program that specifically offers LM services. Such approaches to treatment have been shown to produce dramatic benefits [6] in health outcomes, including weight loss [7,8], cardiometabolic improvements [9], diabetes remission [8], and cardiovascular disease [10,11,12].

Although education on nutrition and healthy lifestyle behaviors delivered during medical training is extremely limited [13], awareness of lifestyle medicine is growing [14] among practitioners due to its superior treatment outcomes [7,9,15]. However, its adoption is hindered by a reimbursement policy that supports traditional fee-for-service payment, incentivizing brief appointments, medications, surgeries, and procedures. Reimbursement is driven by a three-step coding process [16]: (1) the diagnosis is coded using the International Classification of Disease (ICD-10) system; (2) the medical service is coded by current procedural terminology (CPT^®^); and (3) the payment is based on the resources-based relative value scale (RBRVS). Lifestyle medicine (LM) is currently not adequately supported by any of these three processes.

In terms of coded medical services, because LM is founded on health behavior education [17], some of the important components of LM medical services are not acknowledged as reimbursable treatment [18] by the current reimbursement structure [19]. These include structured education programs with healthcare professionals [15], longer appointment times [20,21,22], more frequent follow-up when initiating a lifestyle change [20,21,22], care from an interdisciplinary team [23,24,25], care locations outside of healthcare buildings [15,26], patient group support [21], use of electronic health tools [27,28], and use of demonstration kitchens [18,29]. Reimbursement for health behavior education and intensive support for patient lifestyle change remain limited, and in a previous survey of ACLM members, practitioners reported frustration and an inability to sustain their LM practice in the long-term without appropriate reimbursement, with approximately 57% of ACLM members not receiving compensation for the LM services they provide [18].

The United States (US) healthcare system is predominantly founded on a fee-for-service model, which has incompatibility [30] with delivering care utilizing a patient-centered approach that aligns with LM. A variety of other payment structures exist, including the value-based or risk-based model, shared savings through accountable care organizations, employer-based healthcare plans, health maintenance organizations (HMOs), and preferred provider organizations (PPOs). The World Health Organization (WHO) encourages universal healthcare, emphasizing that many countries employ this approach with better health outcomes at lower costs; these systems more easily facilitate the use of preventative services and lifestyle interventions [31]. This is currently not available within the US. Therefore, it becomes necessary to establish other ways for practitioners and entire healthcare teams to be paid for lifestyle-related evidence-based practices while they navigate the present web of various payer sources. As the public discourse around value-based care [32] and high value care [33] continues to develop, there is a growing need to explore LM as a strategy for producing improved health outcomes more efficiently while maintaining awareness of the potential limitations of patient adherence.

The objectives of this study were to (1) characterize challenges in reimbursement for clinicians who wish to practice LM, (2) present individual comments shared by clinicians in their own words describing their experiences with reimbursement and quality measures, and (3) propose policy changes to better support LM practice as a viable, financially rewarding path for clinicians.

## 2. Materials and Methods

A closed, cross-sectional, survey hosted in the survey platform QuestionPro was administered to N = 3182 members of the American College of Lifestyle Medicine (ACLM) in the summer of 2019. Questions asked covered eight topics: respondent demographics, motivation and interest for practicing LM, medical practice and patient outcomes, reimbursement logistics, quality measures, follow-up on patient outcomes, behavior change and education, and practitioner quality of life. The survey included both multiple-choice and free-text questions. A sample of interest was restricted to consented individuals >18 years, self-identifying as a healthcare practitioner, and completing the survey within the US. The analysis for this paper, including descriptively summarizing quantitative data relating to practitioner demographics and reimbursement, was completed using SAS software, Version 9.4 of the SAS System for Windows, SAS Institute Inc., 2013, Cary, NC, USA. Free-text data were qualitatively coded in Excel by a single researcher (K.F.) and then double checked and collapsed for more parsimonious categories by a second researcher (M.K.). Key quotes were extracted to represent repeating themes. Policy priorities were developed through discussion with ACLM leadership, informed by survey results. This study was approved by the University of New England IRB.

## 3. Results

A total of *n* = 1286 respondents began the survey, and after restricting the survey to those who answered yes to being a healthcare practitioner, and to being over the age of 18, provided informed consent, and responded to the survey from within the United States (based on an Internet Protocol (IP) address), a total sample of *n* = 857 was included in this analysis (Table 1). Due to skipped questions and drop-outs, smaller subgroups of participants responded to specific questions on LM practice (Table 1) and questions on payment and reimbursement (Table 2).

Of this sample, 58% were female, with a median age of 51. Top reported clinical degrees were medical doctor (MD) or doctor of osteopathy (DO) (54%); registered nurse (RN) or registered dietician (RD) or occupational therapist (OT) or physical therapist (PT) (12%); and doctor of nursing practice (DNP) or advanced practice nurse/advanced practice registered nurse (APN/APRN) (4%); other clinical degree (9%); and not selected (22%). The top reported specialties were family medicine (23%) and internal medicine (17%). Of those with a complete answer (*n* = 726), the median number of years in practice was 17. A minority of the sample (17%) reported that all their practice was LM, while 56% reported that some of their practice was LM, and 9% reported that none of their practice was LM.

Of those who responded to the questions on payment, a total of 55% of practitioners reported not being able to receive reimbursement for LM practice. At the same time, almost 60% of practitioners reported that either: (1) their employer or leadership were supportive (20%), (2) the owner of their practice is a LM practitioner (14%), or (3) that there was some or growing support for LM (25%). The single most frequently reported method of payment that did not involve billing insurance was out-of-pocket or direct pay (34%). However, 28% of respondents reported that they were not paid, not well-paid, or did not know how to get paid, and 11% were limited to incorporating LM into existing standard appointments. A total of 59% reported that robust income from shared medical appointments is restricted or a challenge and 33% reported that there are quality measures hindering their practice of LM. When asked what changes would be necessary to make the practice of LM easier, reimbursement overall (18%), reimbursement for more time spent with patients (17%), more support from leadership (16%), policy measures to incentivize health (13%), education in LM for practitioners (11%), LM-specific billing codes and billing knowledge along with better EMR capabilities and streamlined reporting/paperwork (11%), and reimbursement for the extended care team (10%) were among top-reported suggestions.

Direct quotes captured by the free-text fields in the survey related to the themes of barriers to insurance reimbursement, challenges of the time limitations of appointments, perverse incentives for health outcomes improvement, LM made possible through grants or philanthropy, and alternative payment models are presented in Table 3.

## 4. Discussion

Results of this cross-sectional survey describe logistical challenges faced by LM practitioners and those who want to practice in the field, particularly regarding payment and reimbursement. While a majority of survey respondents reported practicing LM for some part of their practice, and while a majority of respondents also reported having some degree of supportive leadership, over half of the respondents reported not being able to achieve reimbursement for the LM services. Support from leadership could occur in a number of ways, including budgetary planning, offering necessary space to practice, and flexing productivity expectations. This result is consistent with a previous survey of LM practitioners in which only 43% of the respondents reported receiving any form of compensation for their LM services. Of those receiving compensation in that survey, almost 44% identified cash payments from patients, philanthropic support, employer-funded programs, and “other” as the source of funding [18]. Although it is admirable that many practitioners are able to work towards creative solutions for payment, this is not feasible for everyone; if reimbursement were better for LM healthcare services, outside funding sources such as these would not be necessary. It is apparent that institutional and professional support for LM approaches has not yet been translated into sustainable reimbursement for these services.

As previously described, LM practice involves multiple elements of care that fall outside the current fee-for-service model, including more time and follow-up with patients [20,21,22], care from an interdisciplinary team [23,24,25], alternative locations for education and delivery of care [15,18,26,29], and use of patient group support [21], and apps [27,28]. Our survey results were consistent with these requirements; when asked what could make the practice of LM easier, suggestions included overall improved reimbursement for LM, reimbursement for increased time with patients, including longer appointments and more frequent follow-up, more support from leadership and colleagues, policy changes to incentivize improved health outcomes such as in value-based care, LM-specific billing codes, and improved reimbursement for other members of the healthcare team and group visits.

Respondents described billing for LM practice as “prohibitive” and “no one knows how”, and described multiple constraints on time available to adequately educate patients in appointments, as well as time to develop materials and programs. These comments are indicative of the paradigm shift required to make healthy lifestyle change the foundation of healthcare. While patients are responsible for their own health behaviors, without adequate teaching time, support, accountability, and all the components of successful behavior change programs, uptake and adherence will be limited. The practitioners surveyed here report seeking creative ways to deliver these elements that will help patients be successful, but in many cases, this results in unpaid work, work that is not well-paid, or work that cannot be billed for because of the site of delivery such as a community location or teaching kitchen. A small segment reported seeking grant funding and philanthropic support to enable their LM practice (“I am currently writing up a research grant to help offset costs” and “so far it’s all been ‘gratis’ on my part”). While admirable from the perspective of social responsibility, such methods of payments are not feasible for most practitioners, who also may have high rates of burnout and have usually entered the workforce with considerable personal debt from student loans [34,35,36,37,38,39], with physicians averaging more than USD 200,000 owed [34,35]. It is unreasonable to expect practitioners who are engaged in evidence-based and effective therapies that align with national guidelines for management of chronic diseases such as obesity, cardiovascular disease, and diabetes to finance their efforts through philanthropy alone.

A minority of respondents reported working with an interdisciplinary team all of the time, and this has implications for the financial sustainability of delivering health behavior change interventions in the emerging context of value-based care. Coaching, counseling, or group education is best administered by professionals trained for those services, not necessarily physicians, because of the cost of service. At the same time, reimbursement for other healthcare professionals, such as RDs, educators, OTs/PTs, and group visits, group programs, and coaching/counseling, were among the top-suggested strategies for making the practice of LM feasible and sustainable. Practitioners also suggested policy changes to incentivize improved health outcomes and prioritize LM approaches; this is the foundation of value-based care. However, while value-based care holds great potential for intersecting with LM practice, mostly because of the superior outcomes made possible by sustained health behavior change, the success of the value-based care model lies in the details. Patient adherence is a critical limitation on the feasibility of paying practitioners based on outcomes, for both LM and conventional medical practice. Without an adequate support structure in the form of a healthcare team, thorough education, sufficient time with educators and practitioners, and referrals to group programs, LM interventions are handicapped from the outset and likely not to work. Intensive therapeutic lifestyle change cannot be effectively delivered in a portion of a 15 min appointment. Investment in staff resources, time with patients to support their health behavior change journey, and nurturing long-term relationships to maintain the desired changes are essential for success. These nurturing practitioner–patient relationships not only support effective behavior change but also improve practitioner satisfaction levels and well-being, with the potential to lower burnout rates.

The authors report that physician residents and other healthcare professionals are hesitant to commit/plan to become certified in LM when there are no reimbursement/payments benefits. There is currently no reimbursement advantage for specialized LM care. While the residents acknowledge the education and training as beneficial, lack of financial gain due to inadequate reimbursement appears to be a hindrance.

While many population-based and episode-based alternative payment models (APMs) [40] have been proposed and implemented in the United States, at present, results from our survey suggest that their effectiveness for LM is limited. The population-based models require practitioners to take on the majority of financial and clinical risk for a group of patients, while with episode-based models, payment incentivizes practitioners to provide high quality, low-cost care to a select group of patients, such as those requiring joint replacements or cancer care. The top reported strategy for LM payment outside of insurance was direct pay/out-of-pocket/concierge/membership, a result reflective of the potential for insufficient reimbursement to contribute to health disparities. Patients who can afford LM through private pay, concierge medicine, or employer-funded reimbursement models are able to receive it, while those patients without the financial means to pay out-of-pocket for LM interventions are left without access. Another drawback of APMs of risk-based models is that practitioners may be more willing to select healthier patients and avoid those who are likely to be costly, which furthers health inequity [41].

Our survey results also highlight the reimbursement obstacles and perverse quality measure incentives (greater reimbursement for procedures, surgeries, and medication management, along with penalties for deprescribing medications and insufficient reimbursement for lifestyle counseling) that encourage sick care and discourage genuine health care. Multiple survey respondents indicated that they had been reprimanded or penalized financially for not prescribing certain medications linked to quality measure payments. The results reported here are consistent with a review of more than 1100 National Quality Forum Quality Measures [42] related to common chronic diseases that found that the majority of measures focused on care processes rather than clinical outcomes. For example, many quality measures reward the prescribing of a statin medication for patients with cardiovascular disease or diabetes, without allowance for an alternative quality measure for patients who achieve optimal lipid levels through lifestyle choices. For these quality measures, the prescription of a statin is sufficient for meeting the requirement—there is no further evaluation of whether the patient achieved an optimal outcome with the drug.

There are minimal quality measures that reward the time-intensive interventions necessary for successful LM approaches, such as assessing lifestyle behaviors proven to impact chronic disease progression and intensive therapeutic lifestyle change programs to support patients in sustaining beneficial change. Shared medical appointments (SMAs) can be a way to better utilize time by seeing patients in a group setting and having the opportunity to be reimbursed feasibly. However, the authors report that there are many hindrances to SMAs. It may be challenging to fill the appointment slots. Patients prefer to be seen during the evening or on a Saturday, which makes it difficult to count on filling daytime group visits with a targeted minimum number. The authors’ experience was consistent with the survey results, showing that most respondents found that SMA reimbursement was restricted or challenging. Location of service is also a barrier for SMAs, as the payor may deny a group visit claim if the location is lacking a National Provider Identifier (NPI) number. Thus, while these SMAs do have potential, more work needs to be done to ensure that the reimbursement is sustainable.

Quality metrics that focus on care processes instead of outcomes inherently overlook patient choice, shared decision making, person-centered care [43,44], and provider well-being, while adding more administrative burden on the practitioners without improving the patient experience or lowering the cost of care. Along with these misaligned quality measures, the Medicare Risk Adjustment with Hierarchical Condition Categories (HCC) coding for Medicare Advantage (MA) plans and Medicare Shared Savings Program (MSSP) Accountable Care Organizations (ACOs) financially penalize clinicians when their patient outcomes are superb since practitioners receive less reimbursement when the patient’s condition improves, thereby putting them in a lower risk category with a lower capitation rate or lower benchmark for expenditures, which impacts shared savings calculations [45].

The current healthcare quality measures, performance measures, and incentive models are tied to a “disease management” model. Improvement in biometrics and resolution of symptoms to the point of no longer needing medication is not acknowledged as a primary goal, or even an option, within current reimbursement models. Misaligned quality measures in which the majority of measures focus on care processes instead of patient outcomes require re-examination.

While much work remains to improve reimbursement for lifestyle interventions, a few models do exist. Some of the first lifestyle-based medical treatment programs to be reimbursed were the intensive cardiac rehabilitation programs (ICRPs) and the Medicare Diabetes Prevention Programs (MDPPs). These programs offer lifestyle change opportunities within a group setting with demonstrated effectiveness [11,46,47,48]. In August of 2010, the Centers for Medicare and Medicaid Services (CMS) made the decision to provide Medicare coverage for ICRPs [49,50]. However, despite coverage, these programs are still not widely available to Medicare recipients and tend to be underutilized [51]. The situation for MDPPs is similar, although there are current policy efforts to increase program numbers, the number of participants, and reimbursement for services [52]. Currently, a gap remains with respect to policy supporting LM practice and payment. For example, the 2020 MDPP billing and payment fact sheet [53] lists a maximum reimbursement of USD 468 for the entire first year for patients who attend 20 one-hour sessions over the course of 12 months and lose/maintain at least a 5% weight loss by the end of the year. In contrast, a physician could bill for over USD 1200 of reimbursement in just one morning of outpatient clinic caring for individual patients utilizing the standard 99213 CPT code [54], regardless of the patient outcome for those visits. The financial incentives are absurdly misaligned with desired outcomes; especially considering that the Diabetes Prevention Program has been shown to decrease the risk of progression to type 2 diabetes in patients with impaired glucose tolerance by 58% at 4 years of follow-up, a significant improvement compared with both standard care and treatment with metformin [55].

Policy changes intended to facilitate sustainable LM practices that address both the barriers to LM practice and the limitations of current payment and reimbursement models e are proposed in Table 4. Some solutions can be implemented locally or within a company, while others might require state-level or national buy-in and should be considered as possible targets for advocacy efforts. These policy proposals relate to three areas of focus:

1. Support for the *care process using an LM approach*, specifically the six pillars of LM: a whole-food, plant-predominant diet, physical activity, restorative sleep, stress management, positive social connection, and avoidance of risky substances;
2. Reimbursement emphasizing outcomes of health, patient experience, and person-centered care;
3. Incentivizing treatment that produces substantial outcome improvements vs. conventional care, such as outcomes of disease remission/reversal.

Additionally, identifying policy improvements may not be enough; decision-makers need to truly understand the benefits of lifestyle medicine to impact care and payment. They may also need education about what LM is and what it looks like; understanding the needs and constraints of a different practice structure is necessary for effectively supporting a different reimbursement structure. This is a barrier to implementing practice and payment reform. In addition to these policy changes, effective communication strategies are needed to disseminate research results to a broad array of stakeholders. To successfully influence policies in reimbursement and payment models that support LM, the education of decision-makers is vital [61].

A strength of this study is that it is one of the first surveys to describe logistical challenges with LM practice, using practitioners’ own words. A limitation of this study is that it is a cross-sectional survey only, and therefore, results should not be over-interpreted. Additionally, the sample was self-selected from within the closed list of ACLM members, and there was a high dropout rate. More research is needed to better characterize the demographics of LM practitioners, such as medical specialty, the extent to which individuals practice LM, and their associations with payment barriers and successes. Research on health outcomes comparing LM to standard medical care is required, along with better reimbursement, to enable benchmarking and comparisons of the value of LM care with conventional treatment. Additionally, further research on improving LM reimbursement is needed, such as case series that incorporate in-depth interviews, simulations of reimbursement, and randomized controlled trials comparing various payment models for health outcomes and effective use of resources. Beyond research, policy changes are necessary to implement the suggestions outlined here.

LM promotes core values of public health in terms of supporting health behaviors, but it adds a novel dimension to clinical healthcare that has traditionally focused on symptom management. For LM to be effective in achieving meaningful health improvements, individuals need customized LM services delivered at the appropriate therapeutic dose [62], in the same way that pharmacotherapy and other treatments are appropriately dosed for the target condition. While some have argued that population health has been medicalized [63], clinical population health standards do not currently encompass health behaviors of the patient. Integrating patient behavior targets into the standard of care will bring public health closer to clinical practice and will enable better testing of LM treatment. This allows for the benchmarking of best LM practices and quantification of benefits of LM, which is critical for intelligently adjusting reimbursement. Urgent attention to defining the financial worth of an improved health outcome is necessary for creating a fair reimbursement model for practitioners who offer real healthcare instead of simply managing sick care.

## 5. Conclusions

The results of this research have demonstrated the reimbursement barriers faced by healthcare practitioners who implement lifestyle medicine within their practices. While chronic disease management guidelines are all quite clear in recommending health behavior changes, the health systems and policies needed to support these interventions are lagging. If optimal health outcomes for chronic diseases truly are the goal, then significantly more resources need to be mobilized to best illuminate what types of lifestyle interventions are most impactful and how to successfully implement them in a sustainable manner. Benchmarking for best LM practices and quantification of benefits of LM will help to support further reimbursement. Now is the time to acknowledge that chronic diseases such as cardiovascular disease, diabetes, and obesity are reversible, that remission is possible, and that LM practitioners who efficiently address the mitigating factors of disease to achieve these outcomes should be paid for their services in a manner that is commensurate with the value they provide, thereby further promoting the growth and accessibility of LM interventions.

## Figures and Tables

**Table 1:** Characteristics of lifestyle medicine practitioners (*n* = 857).

Variable	Median (SD)
**Age (median, SD)**	51 (12)
**Number of years in practice median (SD)** ^4^	17 (11)
**Variable**	**%**
**Gender (% female)**	**58** ^1^
**Clinical Degree**	
MD or DO	54
DNP or APN/APRN	4
RN or RD or OT or PT	12
Other clinical degree	9
Not selected	22
**Specialty** ^2,3^	
Family Medicine	23
Internal Medicine	17
Obstetrics and Gynecology	3
Pediatrics	5
Preventive Medicine	5
Other	23
Not boarded by an ABMS board	21
**Currently practicing LM** ^5^	
Yes—for some things	56
Yes—all my practice is LM	17
No—not at all	9
Not answered	16
**Are you certified in LM?** ^6^	
Yes	20
No	63
Not answered	13
**Proportion of patients being given LM treatment** ^7^	
All my patients	20
Most of my patients	22
About half of my patients	7
Some of my patients	26
None of my patients	2
Not answered	19
**Percentage of the time work within an interdisciplinary** ^8^ **team**	
100	19
75	11
50	12
25	26
Never	9
Not answered	20
**Other team members** ^2,9^	
Physician	41
Nurse practitioner or registered nurse	37
Physician Assistant	14
Dietitian	32
Physical therapist	24
Exercise physiologist	10
Occupational therapist	8
Chiropractor	6
Health coach	16
Massage therapist	7
Other allied health professional	19

Note: Abbreviations include medical doctor (MD), doctor of osteopathy (DO), registered nurse (RN), registered dietician (RD), occupational therapist (OT), physical therapist (PT), doctor of nursing practice (DNP), advanced practice nurse/advanced practice registered nurse (APN/APRN), American Board of Medical Specialties (ABMS). ^1^ *n* = 7 prefer not to answer (1%); ^2^ Multiple answer checkboxes. Percentages do not add up to 100%. ^3^ *n* = 12 prefer not to answer (1%). ^4^ Total *n* = 726 with completed answer. Recoded from free-text answers. Whole number answers were kept the same. Coded “30+” as 30, “0–1” as 1, “25+” as 25, “2.5” as 3, and “PGY-3” as missing. Calculated sum for those who provided two numbers for different positions. ^5^ *n* = 20 prefer not to answer (2%). ^6^ *n* = 35 prefer not to answer (4%). ^7^ *n* = 36 prefer not to answer (4%). ^8^ *n* = 25 prefer not to answer (3%). ^9^ *n* = 24 prefer not to answer (3%).

**Table 2:** Practitioner-reported experiences with payment, quality measures, and suggestions for change.

Variable ^1^	%
**Able to receive reimbursement for LM practice** ^2^ **(%)**	
Yes, at least some LM practice	27
Yes, for all my LM practice	18
No, not for anything	55
**Support from organization leadership for LM practice** ^3,4^	
Employer/leadership is supportive	20
Owner of practice is LM practitioner	14
Some support/support is growing for LM practice	25
Limited/little support for LM practice	18
No support for LM practice	10
Time, productivity, or reimbursement constraints hamper support	6
Disadvantaged populations/uninsured issues pose challenges	2
N/A or no data	3
**Methods of reimbursement other than insurance billing** ^4,5^	
Out-of-pocket/direct pay/concierge/membership	34
Not paid/not well-paid/do not know how to get paid or reimbursed	28
Limited to integrating LM into standard appointments/billing	11
Volunteer/pro bono/grants or community support	6
Salaried/paid by health system or hospital	4
Shared medical appointments	3
Other/NA	12
**Rating of ability to generate robust income through shared medical appointments** ^6^	
Income is robust	6
Income is moderate	36
Income is restricted or a challenge	59
**Are there specific quality measures that are hindering your ability to practice LM?** ^7^	
Yes	33
No	67
**Changes suggested to be necessary to make the practice of LM easier** ^4,8^	
Reimbursement for LM treatment (nonspecific)	18
Reimbursement for increased time spent with patients (longer appt. times; more follow-up visits)	17
Support from leadership and awareness/respect among healthcare practitioners	16
Policy changes to incentivize improved health outcomes; value-based care; prioritize LM approaches	13
Education/training in LM for practitioners	11
LM-specific billing codes and billing knowledge; better EMR capabilities; streamlined reporting/paperwork	11
Reimbursement for extended care team (RDs, educators, OTs/PTs, other health and wellness services, etc., in addition to MD/DOs)	10
Reimbursement for group visits, group programs, educational visits, and coaching/counseling	9
Culture shift to recognize the benefits of LM; education of the public	8
Need more LM research, guidelines, tools, and resources	4
Miscellaneous	8

Note: Abbreviations include not applicable (N/A), electronic medical record (EMR), registered dieticians (RDs), medical doctor (MD, doctors of osteopathy (DOs). ^1^ Total responses vary due to survey drop-out; see footnotes for individual n. ^2^ *n* = 451 total with response; *n* = 117 prefer not to answer; *n* = 289 missing; percentages are out of total with response. ^3^ *n* = 458 total with response; *n* = 110 prefer not to answer; *n* = 289 missing; percentages are out of total with response. ^4^ Coded from free text answers coded with multiple codes; percentages may not add up to 100%. ^5^ *n* = 396 total with response; *n* = 164 prefer not to answer; *n* = 297 missing; percentages are out of total with response. ^6^ *n* = 123 total with response; *n* = 46 prefer not to answer; *n* = 688 missing; percentages are out of total with response. ^7^ *n* = 423 total with response; *n* = 114 prefer not to answer; *n* = 320 missing; percentages are out of total with response. ^8^ *n* = 471 total with response; *n* = 89 prefer not to answer; *n* = 297 missing; percentages are out of total with response.

**Table 3:** Key quotes from practitioners regarding dynamics of LM reimbursement.

Multiple Barriers to Insurance Reimbursement Exist
*“No one knows how. I am billing patients for their annual physical and am incorporating LM into that visit. It has added about 10 minutes to my visit so I am constantly running behind my schedule.”* *“I opened a very small practice with the intention of having extended visits with patients focused on lifestyle medicine. Given the length of the visits and that I am not also doing primary care medicine, I do not think I could get reimbursed by insurance.”* *“The time consumed chasing reimbursement is prohibitive. Even with professional office staff attempting RDN billing, for me, it has proven unsuccessful.* *“Unaware of how to get reimbursed. We are a federally funded health care clinic. Don’t know how LM would be reimbursed by insurance.”* *“It has to be covered by insurance.”*
**Time Limitations on Appointments Pose Challenges**
*“(Need) better reimbursement so that physicians take the time to actually teach LM appropriately.”* *“Time constraints.”* *“More time (needed) for program development, leadership education, marketing, changes in productivity measurements (# patients per day or billable units), ability to hold appointments or group sessions offsite from the hospital/clinic.”* *“(Need) more time for appointments.”*
**Reimbursement and Insurance Models Have Perverse Incentives for Wellness**
*“If my patients are not on ACE inhibitor, statins, DM drugs my ranking goes down, my pay goes down. This makes it very hard to keep doing this work because I am making less money since I will not prescribe the drugs when they are not necessary.”* *“The HEDIS measures are a problem. It is a requirement that patients are on statins if they have CAD (carotid artery disease) or DM.”* *“We have been downgraded in ranking for not prescribing statins, ace inhibitors in individuals managing their DM when they have good A1C’s and are managing their disease through lifestyle.”* *“I did not meet quality measures for heart patients on statins with my ACO because I had many patients coming to my practice because they could not tolerate statins and were referred to me to work on diet.”* *“A Medicare patient was denied coverage for weight management (nutrition) program in favor of a surgical procedure.”* *“DM quality metrics to be on 4 drug regimen—metformin, statin, aspirin, and ace inhibitors. Despite the patient being controlled without, my compensation from hospital was penalized.”* *“Reducing A1C but being penalized for medication non-adherence.”* *“I have received warning letters from insurance companies when my patients were not prescribed recommended drugs.”* *“If we do not have a certain percentage of our CAD patients on a statin medication, my annual bonus gets reduced.”*
**LM Practice Made Possible Through Philanthropy**
*“My appointments are primarily vascular surgery based. I get nothing for the additional time spent counseling on LM.”* *“We are a free health clinic serving clients from 0–200% above the poverty line excepting those eligible for Medicare/Medicaid/ACA. We operate with grants and monetary or in-kind donations.”* *“No one expects a cardiothoracic surgeon to operate with only volunteers and community health workers to assist in the procedure…”* *“I am currently writing up a research grant to help offset costs.”* *“So far it’s all been “gratis” on my part.”* *“I am a volunteer to the community.”* *“Gifted funding by endowment.”*
**Some LM Payments Made Possible by Alternative Models**
*“Our functional medicine/lifestyle medicine practice does limit insurance-based practice currently and is rapidly moving away from an insurance-based model. We can practice better medicine that provides more value to patients outside of an insurance model with far less overhead.”* *“Grants, donations, corporate sponsorships, client self-pay and always LOTS of fundraising and volunteer time.”* *“Health savings accounts, direct pay and membership programs.”* *“I have a direct pay practice, so my patients either pay for a one-off or occasional consults, or (most commonly) patients pay a monthly membership and LM is included.”* *“I work for an HMO and get paid no matter what.”*

Note: Quotes are written as submitted and have not been edited for grammar or syntax. Abbreviations include registered dietary nutritionists (RDN), angiotensin converting enzyme (ACE), carotid artery disease (CAD), diabetes mellitus (DM), Healthcare Effectiveness Data and Information Set (HEDIS), glycated hemoglobin laboratory test levels (A1Cs), Accountable Care Organization (ACO), Affordable Healthcare Act (ACA), Health Maintenance Organization (HMO).

**Table 4:** Policy recommendations to support lifestyle medicine reimbursement and practice.

Area of Focus	Recommendation	Rationale	Potential Barrier(s) or Limitation(s)	Employer/Health SYSTEM/LOCAL Level	State Level	National Level
Care process using an LM approach	Develop new quality measures that focus on the care process using the pillars of LM (i.e., optimal nutrition, encouragement of activity/exercise, restorative sleep, avoidance of risky substances, healthy ways to deal with stress, positive social interactions) and those that emphasize clinical outcomes and patient experience to address chronic disease remission and reversal instead of just chronic disease management.	LM offers powerful interventions for chronic disease management. LM fits into the CMS Meaningful Measures 2.0 plan to modernize healthcare with an emphasis on person-centered care, safety, equity, chonic conditions, seamless care coordination, affordability and efficiency, wellness and prevention, and behavioral health [56]. This new plan is being designed to utilize only quality measures of highest value while alignining these measues across all healthcare stakeholders [56].	Challenges quantifying LM interventions (dietary pattern, activity time/week, referrals for sleep, loneliness, risky substance use).		X	X
Care process using an LM approach	Increase funding for modalities that address patient education and healthcare coaching.	Health behavior education is impossible to effectively deliver in sporadic, brief appointments.	Need workforce trained in LM to execute.	X	X	X
Care process using an LM approach	Incentivize individuals to incorporate intensive LM practices into their lives.	Intensive LM practices lead to both short- and long-term health benefits. Short-term improvements in health help to reinforce behavior change.	Resistance to change.	X		
Care process using an LM approach	Incorporate LM into public health messaging.	Education, increased awareness; “normalizing” LM as an approach for managing chronic disease.	Funding and buy-in.	X	X	X
Care process using an LM approach	Secure reimbursement for innovative LM approaches such as shared medical appointments (SMAs).	Encourage cost-effective, innovative care.	Provider and staff knowledge regarding running SMAs and participants’ concerns regarding “sharing” their medical appointment with others.		X	X
Care process using an LM approach	Encourage electronic health record (EHR) adoption of lifestyle medicine interventions and metrics.	Provide accurate, up-to-date, and complete information and documentation.	EHR limitations, cost.	X		
Care process using an LM approach	Provide funding for lifestyle medicine education for healthcare provider students, including medical, nurse practitioner, and physician assistant students.	Evidence-based, cost effective care.	Availability of LM preceptors/faculty and programs in which to learn.	X	X	X
Care process using an LM approach	Remove specific location billing requirements.	Allows LM programming to be offered where it is accessible to the individuals, such as places of worship, schools, and community centers.	Difficulty changing “status quo”, developing new billing process.			X
Care process using an LM approach	Within values-based reimbursement, consider monthly payments for wellness care and chronic disease management, plus episodic payments for new acute issues [57].	Allows for buffer from unexpected acute episodes that may be outside of the control of the healthcare provider and system.	This would require payors to take on more of the financial risk.	X	X	X
Care process using an LM approach	Reform medical malpractice so that “defensive medicine” is no longer a necessity [58].	Low-value services would be less likely to be ordered and the risk of offering LM from a malpractice standpoint would be decreased.	Medical malpractice reform is a significant undertaking, yet is an important component to improving current medical practice in the United States, including the ability to provide high value services such as LM.		X	X
Outcomes of health, patient experience, and person-centered care	Decrease funding for modalities that are inferior to LM. Scrutinize and refine policies that have led to perverse incentives	Removal of perverse incentives, lower costs, improved outcomes.	Difficulty changing “status quo”, some unwilling to adopt LM techniques.		X	X
Outcomes of health, patient experience, and person-centered care	Ensure that established effective lifestyle medicine programs such as the Diabetes Prevention Program and intensive cardiac rehabilitation are covered by all insurers for all ages, at reimbursement rates that reflect the value of the service offered and the time necessary to effectively provide the intervention.	Evidence-based, cost-effective care.	Availability of these programs, current inadequate reimbursement for the National Diabetes Prevention Program (DPP).		X	X
Outcomes of health, patient experience, and person-centered care	Pass cost savings onto the provider or consumer.	Financial incentives may help drive patient behavior change and provider practice style.	Difficulty quantifying.		X	X
Outcomes of health, patient experience, and person-centered care	Require informed consent for chronic disease surgeries and procedures to include viable lifestyle medicine options when these are appropriate. One example would be for those experiencing cardiac symptoms and diagnoses who could benefit from intensive cardiac rehabilitation and traditionally are offered medications, stents, and/or surgery. Mandate that the benefits of intensive lifestyle changes be a part of informed consent prior to procedures, medications, and surgeries for diseases in which the evidence demonstrates safety and efficacy for lifestyle changes.	Informed consent is the legal and ethical basis for making certain the individual is fully informed regarding options and the chosen intervention [59].	Time, effort, increased complexity of decision making.	X	X	X
Outcomes of health, patient experience, and person-centered care	Inject Health in All Policies [60] framework at the local, state, and national level whenever new policies regarding healthcare reimbursement are proposed.	Encourages health, equity, and sustainability.	Difficulty changing culture with respect to the role of medical care and insurance.	X	X	X
Incentivize disease remission/reversal	Revise reimbursement to reflect that individuals do have the ability and option to reverse their non-communicable chronic disease instead of sticking with the assumption that all chronic diseases only progress and require further medical interventions.	Offers reimbursement for LM and recognition for high-value and improved outcomes.	Difficulties quantifying how to measure reimbursement.		X	X
Incentivize disease remisison/reversal	Encourage LM initiatives and reimbursement within government payer models, including Medicaid and Medicare, as well as a public option if this becomes available.	Offers reimbursement for LM and recognition for high-value and improved outcomes.	Concerns regarding noncompliance.		X	X
Incentivize disease remisison/reversal	Provider incentives based on high-value services and improved health outcomes as compared to medications prescribed.	Offers reimbursement incentives for LM and recognition for high-value and improved outcomes.	Will require a monumental shift in what types of care are emphasized and delivered.		X	X

Note: Abbreviations include Centers for Medicare & Medicaid Services (CMS), electronic health records (EHR), Diabetes Prevention Program (DPP).

## Data Availability

Data used in this survey is not publicly available.
